# Cell Culture Models for the Investigation of Hepatitis B and D Virus Infection

**DOI:** 10.3390/v8090261

**Published:** 2016-09-20

**Authors:** Eloi R. Verrier, Che C. Colpitts, Catherine Schuster, Mirjam B. Zeisel, Thomas F. Baumert

**Affiliations:** 1 Inserm, U1110, Institut de Recherche sur les Maladies Virales et Hépatiques, 3 rue Koeberlé, 67000 Strasbourg, France; e.verrier@unistra.fr (E.R.V.); colpitts@unistra.fr (C.C.C.); catherine.schuster@unistra.fr (C.S.); mirjam.zeisel@unistra.fr (M.B.Z.); 2 Université de Strasbourg, 4 rue Blaise Pascal, 67081 Strasbourg, France; 3 Institut Hospitalo-Universitaire, Pôle Hépato-digestif, Nouvel Hôpital Civil, 1 place de l'hôpital, 67000 Strasbourg, France

**Keywords:** Viral cell entry, hepatocytes, hepatoma cells, life cycle, NTCP

## Abstract

Chronic hepatitis B virus (HBV) and hepatitis D virus (HDV) infections are major causes of liver disease and hepatocellular carcinoma worldwide. Despite the presence of an efficient preventive vaccine, more than 250 million patients are chronically infected with HBV. Current antivirals effectively control but only rarely cure chronic infection. While the molecular biology of the two viruses has been characterized in great detail, the absence of robust cell culture models for HBV and/or HDV infection has limited the investigation of virus-host interactions. Native hepatoma cell lines do not allow viral infection, and the culture of primary hepatocytes, the natural host cell for the viruses, implies a series of constraints restricting the possibilities of analyzing virus-host interactions. Recently, the discovery of the sodium taurocholate co-transporting polypeptide (NTCP) as a key HBV/HDV cell entry factor has opened the door to a new era of investigation, as NTCP-overexpressing hepatoma cells acquire susceptibility to HBV and HDV infections. In this review, we summarize the major cell culture models for HBV and HDV infection, discuss their advantages and limitations and highlight perspectives for future developments.

## 1. Introduction

Chronic infection by hepatitis B virus (HBV) is a leading cause of liver disease worldwide. An estimated 250–300 million patients are chronically infected by the virus, which is responsible for progressive liver disease and liver failure as well as hepatocellular carcinoma (HCC), the second deadliest form of cancer [1,2,3,4]. Although an efficient vaccine is available, the global prevalence of HBV infection has remained virtually unchanged. Current licensed therapies based on nucleos(t)ide analogs (NUCs) and pegylated type-I interferon (IFN) effectively control viral replication and reduce progressive liver disease. However, viral eradication remains extremely rare in chronically infected patients [5,6].

**Figure 1:**
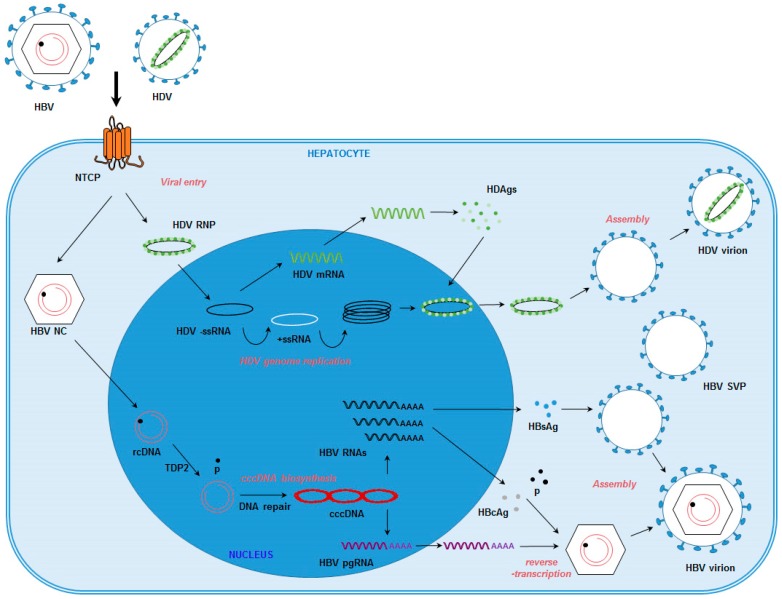
Schematic representation of the HBV and HDV life cycles in hepatocytes. HBV: hepatitis B virus; HDV: hepatitis D virus; HDV RNP: HDV ribonucleoprotein. HBV NC: HBV nucleocapsid. rcDNA: relaxed circular DNA. −/+ssRNA: negative/positive single-stranded RNA. cccDNA: covalently closed circular DNA. HBV pgRNA: HBV pregenomic RNA. HDAgs: hepatitis D antigens. HBcAg: HBV core antigen. HBsAg: HBV surface antigen. p: HBV polymerase. HBV SVP: HBV subviral particle. TDP2: tyrosyl-DNA phosphodiesterase 2.

HBV is a small enveloped DNA virus belonging to the *Hepadnaviridae* family [4], which exclusively infects human hepatocytes [4]. HBV virions (also known as Dane particles) are enveloped particles approximately 42 nm in diameter, comprised of the nucleocapsid, which incorporates the HBV core antigen (HBcAg), and the viral genome, a 3.2 kb partially double-stranded relaxed circular DNA (rcDNA) [7,8]. Embedded in the viral envelope are three proteins: small S (S), middle M (S + preS2 region) and large L (S + preS2 region + preS1 region), forming the HBV surface antigen (HBsAg) [9]. The presence of these viral proteins in the infected cells allows for detection of HBV infection by enzyme-linked immunosorbent assay (ELISA) [10] or immunofluorescence [10,11,12,13]. HBV infection of hepatocytes is initiated by low-affinity attachment to heparan sulfate proteoglycans (HSPGs), including glypican 5 (GPC5) [12]. Subsequent specific binding to the sodium taurocholate co-transporting polypeptide (NTCP) (Figure 1), a hepotocyte-specific receptor, induces entry of the viral particle into its target cells (reviewed in [14]). Following delivery of the viral genome into the nucleus of infected cells, HBV rcDNA is converted into covalently closed circular DNA (cccDNA). This replication intermediate allows the transcription of viral RNAs, including HBV pre-genomic RNA, which is then reverse-transcribed into nascent genomic DNA by the viral polymerase [15]. The cccDNA is responsible for viral persistence in infected hepatocytes and the establishment of chronicity [15]. HBV DNAs and RNAs can be detected by Southern blot and Northern blot, respectively, or by quantitative PCR (qPCR) methods [10,11,16]. cccDNA production induces the expression of the soluble HBeAg, which can be detected by ELISA [10,11,16]. Current therapeutic strategies do not prevent the formation of cccDNA or lead to its eradication. Therefore, treatment does not usually result in viral clearance, even though viral infection can be controlled if treatment is administered for life [15].

In the liver, HBV-infected hepatocytes can be co-infected with the hepatitis D virus (HDV), a satellite virus of HBV. HDV, a small RNA virus that resembles plant viroids, uses the HBV envelope proteins for assembly of its infectious particles. Consequently, HDV and HBV are expected to share the same receptors at the surface of hepatocytes and the same entry pathway [14]. Therefore, cell lines that are permissive for HBV infection will also support HDV entry. The HDV genome is a circular negative single-stranded RNA encoding one structural protein, the delta antigen (HDAg) [17]. HBV/HDV co-infections are thought to be responsible for increased liver damage and higher HCC risk compared to HBV mono-infections [18]. To date, no efficient curative treatment for HDV infection is available [19].

Since current antiviral treatments are unable to induce viral clearance, new therapeutic strategies are urgently needed. One reason for the current dearth of curative treatments is the limited understanding of virus-host interactions, which has been hampered by the absence of robust cell culture models supporting viral infection. The recent discovery of NTCP as a bona fide receptor for the two viruses [10,11] has opened new perspectives for a better understanding of key steps of the viral life cycle, which may also serve as targets for the cure of infection. Here, we review the cell culture models available for HBV and HDV infections.

## 2. Primary Human and Tupaia Hepatocytes

Human hepatocytes are the exclusive hosts of HBV and HDV. For a long time, primary cultures of human hepatocytes (PHH) were the only suitable in vitro model for the study of HBV infection [20]. However, studying HBV/HDV infection in PHH is limited by a series of constraints. First of all, PHH usually do not expand in culture, have a limited life span of weeks, and are difficult to manage in culture conditions [4]. Additionally, their supply is limited [4]. Moreover, infectivity of HBV for PHH drops rapidly after their plating, due to the loss of hepatocyte polarization under culture conditions [20]. Finally, in addition to restrictive culture conditions (including the use of dimethyl sulfoxide—DMSO), the infection of PHH by HBV is strongly dependent on the genetic background of the host. Consequently, high donor-to-donor variability is observed, limiting the number of reproducible studies [4,21]. The recent development of novel technologies allowing the long-term culture of primary hepatocytes may help to provide a more stable system for studying viral infection [22]. Interestingly, HBV and HDV can also infect primary cultures of *Tupaia belangeri* hepatocytes (PTH) [23,24]. This tree shrew hepatocyte model, used by Yan and colleagues [11], enabled the identification of NTCP as a receptor for HBV and HDV, demonstrating the suitability of PTH for the study of HBV and HDV infection. Furthermore, this model has been useful to investigate the mechanism of action of antivirals [25] as well as the phenotype of HBV variants isolated from patients [26]. Despite their limitations, primary cultures of hepatocytes are still the most physiologically relevant in vitro model for the study of HBV and HDV infection, presenting typical characteristics of the natural host of these viruses, such as hepatocyte polarization and the comprehensive presence of hepatic host factors. Moreover, they exhibit a fully functional innate immune system, allowing the study of the sensing of the virus and the antiviral response to HBV infection [27,28,29]. As such, they are widely used to validate HBV/HDV-related host factors and to confirm the activity of antiviral compounds [10,11,12,30,31].

## 3. HepaRG Cell Line

The HepaRG cell line is a hepatic progenitor cell line derived from a hepatitis C virus (HCV)-induced liver tumor [32]. In contrast to other liver cancer–derived cells, HepaRG cells maintain a high number of physiological hepatic functions and demonstrate a transcriptomic pattern more closely resembling that of hepatocytes. In particular, the cytochrome P450 expression level and expression of innate immune components reflect that of PHH [28,33]. As such, they are extensively used for drug metabolism and toxicology assays [32]. Interestingly, following a long-term DMSO-mediated differentiation process, HepaRG cells differentiate into hepatocyte-like cells and acquire susceptibility to HBV and HDV infection, constituting the first suitable cell culture system for infection assays [34]. In this context, HepaRG/HBV and HDV systems were used to identify small molecules inhibiting HBV infection, such as ezitimibe [35]. Notably, HepaRG cells support both viral entry and production of cccDNA [36] and are thus suitable for the study of a large number of steps in the HBV life cycle.

In 2007, Schulze and colleagues [37] took advantage of the HepaRG cell line to demonstrate the importance of HSPGs in the initiation of HBV entry. Using HepaRG cells and HDV as a surrogate for studying HBV entry, Sureau and Salisse subsequently identified a heparan sulfate binding site in the antigenic loop of the HBV envelope protein as a critical determinant for HBV/HDV infectivity [38]. Given that non-differentiated HepaRG cells do not support HBV/HDV entry, whereas differentiated HepaRG cells do allow HBV/HDV entry, comparative analysis of non-differentiated and differentiated cells is a powerful tool to identify hepatic host factors required for viral infection. In this context, Ni and colleagues [10] compared the transcriptomic patterns of differentiated and naive HepaRG cells to confirm the crucial role of NTCP as a cellular receptor for both HDV and HBV. Finally, although the mechanisms involved in HBV internalization are still unclear, one study used HepaRG cells to indicate the putative role of caveolin-1 in this process [39]. In response to HBV infection, HepaRG cells establish an efficient antiviral immune response that controls HBV replication [40], making this cell line suitable for the study of the innate immune factors involved during HBV infection. In particular, two recent studies in the HBV/HepaRG model demonstrated an early and active inhibition of the innate immune response by HBV, which may at least partially explain the “stealth” behavior of HBV in hepatocytes [28,41]. However, despite the numerous advantages outlined above, the HepaRG infection model remains restrictive. In particular, the cells require a long-term differentiation process which may affect the reproducibility of experiments. Moreover, the infection efficiency is low. Both limitations severely restrict the suitability of this cell line for high-throughput studies.

## 4. Huh7 and HepG2 Cell Lines

Huh7 and HepG2 cell lines are hepatoma-derived perpetual cell lines widely used as a surrogate model for hepatocytes, even if they only partially mimic physiological hepatic functions. In particular, these cells are not susceptible to HBV and HDV infection, as they are unable to mediate viral entry [42,43]. However, these cells do support complete HDV replication, as their co-transfection with plasmids encoding the HDV genome and HBV envelope proteins leads to the production of recombinant HDV virions capable of infecting susceptible cells [12,44]. Furthermore, the transfection of hepatoma cells with replication-competent HBV DNA or circular HBV DNA triggers the production of HBV particles [12,45,46,47]. Two HepG2-derived cell lines, HepAD38 [48] and HepG2.2.15 [49], were stably transduced with the HBV genome and are commonly used as a source of HBV infectious particles for infection assays [10,12,50]. Interestingly, transduction of the HBV genome is sufficient to induce the production of low levels of cccDNA [51], making this model suitable for the study of many steps in HBV replication, including cccDNA formation and its regulation. Indeed, an elegant study using hepatoma cells as a validation model recently demonstrated that the cellular DNA repair enzyme tyrosyl-DNA phosphodiesterase 2 (TDP2) (Figure 1) was responsible for the dissociation of the viral polymerase from the HBV rcDNA, thus representing a critical step in the initiation of cccDNA formation [16]. Given the ease of use of this model, hepatoma cells have been extensively exploited for the study of virus-host interactions in the past decades [21]. Moreover, as stable replicating systems, HepAD38 and HepG2.2.15 cells are interesting tools for the screening of antiviral molecules [52]. However, due to the lack of viral entry, these models do not allow a comprehensive understanding of the full viral cycle, including the early stages of viral entry and trafficking in hepatocytes.

## 5. NTCP-Overexpressing Hepatoma Cell Lines

NTCP is a bile acid transporter exclusively expressed at the basolateral membrane of hepatocytes [53]. Two independent studies recently demonstrated that NTCP is a specific receptor for both HBV and HDV [10,11], which may also contribute to the internalization and genome delivery of HBV within infected cells [54]. Interestingly, Huh7 and HepG2 cells lack NTCP expression [11], explaining their inability to mediate viral infection. However, the exogenous expression of human NTCP in hepatoma cells confers susceptibility to HBV and HDV infection [10,11], providing the first robust in vitro model to study the full viral life cycles and to probe the underlying virus-host interactions. It is interesting to note that HepG2-NTCP cells appear to be more highly susceptible to HBV than Huh7-NTCP cells, suggesting that additional factors may be required to achieve optimal viral infection [10].

An increasing number of studies take advantage of NTCP-overexpressing cell lines for the discovery of new factors involved in HBV infection, especially viral entry steps. Using HDV as a surrogate for studying HBV entry and NTCP-overexpressing Huh7 cells, GPC5 was identified as an attachment factor for HBV and HDV at the cell surface of hepatocytes [12]. Bouezzedine and colleagues demonstrated that interleukin 6 (IL6) downregulated NTCP expression, leading to a decrease in HBV entry [55]. Finally, DDX3 DEAD-box RNA helicase has been shown to restrict viral replication in a HepG2-NTCP model [56]. Interestingly, NTCP-overexpressing HepG2 cells have been shown to be susceptible to the entry of lentiviral HBV pseudotypes [57].

NTCP-overexpressing hepatoma cell lines have also been used to screen for antivirals targeting cell entry [13,58]. Myrcludex B, a lipopeptide derived from the HBV envelope protein, binds to NTCP and has been shown to inhibit HBV and HDV entry in a large number of in vitro models [10,14,58,59,60,61,62]. Small molecules targeting NTCP, such as cyclosporin A [13,30], irbesartan [63,64] and vanitaracin A [65], have demonstrated antiviral activity against HBV in NTCP-overexpressing cell lines. These novel cell culture systems clearly provide useful tools to improve our understanding of the HBV and HDV life cycles. Moreover, some other cell lines, such as HLCZ01, were shown to support the full HBV life cycle [66]. However, one limitation is that non-differentiated hepatoma cells are distinct from hepatocytes (e.g., chromosomal abnormalities and mutations, different gene expression and metabolic properties). Therefore, new findings should be validated in alternative model systems, including primary hepatocytes or in vivo models which have been reviewed elsewhere [21].

## 6. Conclusions and Future Developments

As we have described here, recent advances in the field have enabled the development of highly useful cell culture models to improve our knowledge of virus-host interactions. Interestingly, all the models support the major infection steps, from viral entry to cccDNA formation, genome replication, and virion assembly. Viral spread, however, has not been observed in these models [21], although constitutive replication of the HBV genome in several HepG2-derived cells allows for the production of infectious viral particles as described above. Furthermore, the level of cccDNA produced in these cells is extremely low [15]. The current HBV infection models require both polyethylene glycol (PEG) during entry (to enhance glycosaminoglycan-dependent binding [37]), and DMSO (to enhance HBV infection) in every cell culture model [59]. Although the comprehensive effect of DMSO on HBV infection is still unclear, it is well known that DMSO induces cellular polarization and NTCP expression in HepaRG cells and PHH [59,67]. Moreover, DMSO enhances HBV replication in HepG2 cells, suggesting multiple mechanisms of action [68].

Despite these advances, these HBV/HDV infection models still suffer from limitations that should be addressed in future research (Table 1). Indeed, the ultimate HBV/HDV infection model still remains to be developed. The infection efficacy of primary hepatocytes is limited and HepaRG cells are difficult to culture, requiring a time-consuming (one month) differentiation process. Although the establishment of NTCP-overexpressing cells was a critical advance enabling the development of high-throughput assays, these cells are nonetheless cancer cells, thus sharing only some characteristics with human hepatocytes. Interestingly, the overexpression of NTCP in non-human cells, such as the mouse AML12 cell line, induced susceptibility to both HDV and HBV infection [69], although these cells do not express human hepatic factors, making them an interesting but limited model system. Moreover, it is important to note that in all models, a high multiplicity of infection (ranging from 100 to 10,000) is still required to reach acceptable infection levels [42]. Furthermore, the use of PEG and DMSO may restrict the understanding of HBV/HDV-specific entry pathways in hepatocytes [10,11,31,58,70]. Finally, the absence of viral spread and inefficient infection of all cells exposed to the virus are still major limitations of this system (e.g., for high-throughput functional genomics approaches, such as pooled loss-of-function screens).

Another important limitation of all infectious model systems is the low production of cccDNA [21]. As cccDNA has a central role in viral replication and the establishment of chronic infection in the liver, cccDNA is a target of choice for the development of new antiviral therapies [15]. To date, cccDNA biology is still poorly understood, and a much better understanding of cccDNA formation and regulation will be required to evaluate its potential as a target for viral cure. In order to do so, the production of a cell culture model exhibiting high levels of cccDNA following HBV infection is mandatory. Interestingly, a duck HBV model, which produces a high number of cccDNA copies, recently enabled the identification of TDP2 as a key player in cccDNA formation [16]. A comprehensive overview of hepatic host factors involved in cccDNA formation and its regulation may help to produce an improved infection system, which exhibits high levels of cccDNA and is suitable for high-throughput functional genomics or small molecule screens [15].

It is expected that further development of these models (e.g., by increasing their robustness, enhancing cccDNA production and enabling viral spread) will finally allow the discovery of novel therapeutic strategies aimed at curing the viral infection. Moreover, new technologies may allow for better cell culture systems. For example, stem cell–derived hepatocyte-like cells which support HBV infection [71] or liver organoid cultures [72] may provide some interesting tools for a better understanding of HBV infection. In particular, the discovery and development of host-targeting or immunomodulatory agents/targets and approaches will greatly benefit from next generation model systems [62,73].

## Figures and Tables

**Table 1:** Cell culture models susceptible for HBV and HDV infection. The respective model systems and their key advantages and limitations are shown.

	Advantages	Limitations
**PHH**	Natural host of the virus	Limited infection efficacy and replication
	Exhibit hepatic functions	Limited supply
	Most physiological	High donor-to-donor variability
**PTH**	Available from animals bred in-house	Limited infection efficacy and replication
	Allow more reproducible infections than PHH	Non-human cells
**HepaRG cell line**	Exhibit some hepatic functions	Requires differentiation
		Delicate culture conditions
		Limited infection efficacy
**HepG2-NTCP cell line**	High reproducibility	Only partially mimic hepatocytes
	Easy access/supply	High MOIs and PEG required for infection
	Efficient and more robust viral infection	Absent spread, very limited cccDNA synthesis

PHH: primary human hepatocytes; PTH: primary Tupaia hepatocytes; PEG: polyethylene glycol; MOI: multiplicity of infection; cccDNA: covalently closed circular DNA.
